# Association of biochemical indicators with multimorbidity in 19,624 older adult individuals with chronic diseases: a study from Jindong District, Jinhua City, China

**DOI:** 10.3389/fpubh.2025.1472415

**Published:** 2025-01-24

**Authors:** Qihuan Yao, Guozhong Chen

**Affiliations:** Department of Health Management, School of Public Health, Hangzhou Normal University, Hangzhou, China

**Keywords:** multimorbidity, chronic diseases, biochemical indicators, older adult people, risk factor

## Abstract

**Background:**

Chronic disease multimorbidity is influenced by multiple factors, but with little knowledge on the impact of biochemical indicators. This study aims to investigate the prevalence of multimorbidity of chronic diseases among older adult individuals in the community, as well as the factors related to biochemical indicators associated with chronic disease multimorbidity.

**Methods:**

The study included 19,624 older adult individuals aged 60 and above in Jindong District, Jinhua City, Zhejiang Province, China. Participants completed a national standardized older adult health examination in the community. Chi-square tests and logistic regression were employed to evaluate the potential factors of biochemical indicators related to multimorbidity of chronic diseases.

**Results:**

The multimorbidity rate of chronic diseases in older adult patients is 70.3%. Each chronic disease coexists with one or more other chronic diseases in over 75% of cases. Among the biochemical indicators, hemoglobin (Hb) (OR = 1.46, 95%CI: 1.13–1.90), white blood cell count (WBC) (OR = 1.25, 95%CI: 1.02–1.54), red blood cell count (RBC) (OR = 1.36, 95%CI: 1.10–1.69), urinary protein (U-PRO) (OR = 1.10, 95%CI: 1.02–1.19), urinary glucose (U-GLU) (OR = 1.44, 95%CI: 1.23–1.67), alanine aminotransferase (ALT) (OR = 1.71, 95%CI: 1.39–2.10), aspartate aminotransferase (AST) (OR = 1.22, 95%CI: 1.05–1.41), creatinine (Cr) (OR = 1.28, 95%CI: 1.16–1.42), uric acid (UA) (OR = 1.36, 95%CI: 1.22–1.51), total cholesterol (TC) (OR = 1.76, 95%CI: 1.59–1.95), triglycerides (TG) (OR = 2.63, 95%CI: 2.46–2.82), low-density lipoprotein cholesterol (LDL-C) (OR = 1.84, 95%CI: 1.60–2.11), high-density lipoprotein cholesterol (HDL-C) (OR = 10.99, 95%CI: 8.12–14.90), and fasting blood glucose (FBG) (OR = 1.89, 95%CI: 1.74–2.05) are associated with the risk of multimorbidity of chronic diseases (*p* < 0.05). Among these, lipid parameters demonstrated the strongest associations with multimorbidity risk, with low HDL-C showing an 11-fold increase and elevated TG a 2.63-fold increase.

**Conclusion:**

This study found that the prevalence of multimorbidity among older adult individuals in this region reached 70.3%. Multiple biochemical indicators were significantly associated with multimorbidity, particularly lipid parameters (low HDL-C and elevated TG), glucose parameters (elevated FBG and positive U-GLU), liver function (elevated ALT), and hemoglobin levels. These findings provide important evidence for research on factors associated with multimorbidity in the older adult population.

## 1 Introduction

Global population aging is one of the most significant demographic transformation trends of the 21st century (1). The “second baby boom” generation in China has now entered the retirement phase (2), with China experiencing a more rapid demographic aging structure transformation compared to most other countries (3). Data from the seventh national census shows that the total number of older adult people aged 60 and above in China in 2020 was 264 million (4), and it is projected to exceed 400 million by 2035, transitioning from a moderately aging society to a long-term severely aging society (5). In response to this significant demographic shift, China has elevated the active response to population aging to an unprecedented national strategic level, highlighting the urgency of addressing this challenge.

Chronic diseases closely related to aging have become the most challenging health threat in China (6). What is more concerning, the phenomenon of older adult people suffering from multiple chronic diseases is widespread and increasing (7, 8). The Chinese expert consensus on the management of older adult multimorbidity (2023) defines multimorbidity of chronic diseases in the older adult as the simultaneous occurrence of two or more chronic health problems in the same older adult individual (9). Compared to a single chronic disease, managing multimorbidity increases the consumption of medical resources (10), complicates diagnosis and treatment, and reduces patients’ quality of life (11), thereby increasing the risk of disability and death among the older adult (12). Each additional disease reduces life expectancy by 1.8 years (1), and increases disease-related expenses to 3–5 times (13), imposing a heavy burden of disease and economic burden on individuals, healthcare providers, and society. Research shows that the majority of potential for reducing disease burden lies in effective preventive measures targeting the older adult (3).

Multimorbidity has emerged as an increasingly prominent health challenge among older adult populations. International research reveals significant regional variations in chronic disease multimorbidity rates: approximately 63.7% of individuals aged 65 and older in the United States (14), with a systematic study across American and European countries indicating a rate of 67% (15). Compared to high-income countries, low- and middle-income countries (LMICs), such as sub-Saharan Africa, exhibit comparable multimorbidity rates with a notable upward trend, currently experiencing a rapid epidemiological transition toward multiple disease coexistence (16). And studies have shown that the prevalence of multimorbidity among older Chinese adults ranges from 40 to 90% (17–20).

Biochemical indicators serve as a crucial tool for assessing individual health status and hold significant importance in multimorbidity research. Existing studies have predominantly focused on the associations between biochemical indicators and single diseases, such as the relationship between chronic kidney disease and blood biochemical indicators (21), chronic kidney disease and lipid parameters (22), and depression and biochemical indicators (23) Research on multimorbidity and biochemical indicators has explored markers including serum alanine aminotransferase (ALT) (24) and glycated Hb (25). Studies have revealed a dose–response relationship between ALT levels and multimorbidity, with the adjusted odds ratio for the fourth quartile being 4.71 (95% CI: 3.56–6.23), indicating a significant association between elevated ALT levels and chronic disease coexistence, independent of other potential risk factors (24). Another study found that, after adjusting for age and body mass index (BMI), glycated hemoglobin A1c (HbA1c) [1.3836 (95% CI: 1.3087–1.4627)] emerged as the most significant predictor of disease burden, demonstrating a positive correlation with the number of chronic conditions (25).

Studies have shown that lifestyle interventions are important strategies for addressing the public health challenge of multimorbidity in LMICs (16). As objective health assessment indicators, biochemical parameters and their association patterns with multimorbidity warrant attention. However, research examining the associations between multiple biochemical indicators and multimorbidity remains limited.

Since 2022, Zhejiang Province has implemented a free health examination system with uniform standards for urban and rural areas, standardizing the basic examination items and frequency. This has improved the quality of residents’ health records, providing a scientific data foundation for investigating the associations between multimorbidity and comprehensive biochemical indicators among older adults. Therefore, this study aimed to evaluate the associations between multiple biochemical indicators and multimorbidity using health examination data of older adults from Jindong District, Jinhua City, Zhejiang Province in 2022. Through this research, we expect to provide potential insights for designing prospective and intervention studies based on biochemical indicators, offer new perspectives for health management research of older populations in LMICs, and lay the groundwork for exploring methods to identify high-risk populations.

## 2 Materials and methods

### 2.1 Study population

In 2022, a total of 14 township health centers or community health service centers in Jindong District, Jinhua City were selected. Health examination records of older adult individuals aged 60 and above were collected. After excluding missing data, a total of 19,624 complete health examination records were included in this cross-sectional study.

### 2.2 Study variables

The basic information of the study population includes gender, age, and place of residence. The biochemical indicators include the following items: blood routine, which consists of Hemoglobin (Hb), White Blood Cell Count (WBC), Platelet Count (PLT), and Red Blood Cell Count (RBC); urine routine, which includes Urinary Protein (U-PRO), Urinary Glucose (U-GLU), Urinary Ketones (U-KET), Urinary Occult Blood (U-BLD), and Urinary Leukocytes (U-LEU); Fasting Blood Glucose (FBG); liver function indicators, comprising Alanine Aminotransferase (ALT), Aspartate Aminotransferase (AST), and Total Bilirubin (TBIL); renal function indicators, which consist of Creatinine (Cr), Blood Urea Nitrogen (BUN), and Uric Acid (UA); and blood lipid indicators, including Total Cholesterol (TC), Triglycerides (TG), Low-Density Lipoprotein Cholesterol (LDL-C), and High-Density Lipoprotein Cholesterol (HDL-C). Biochemical indicators were tested by trained medical professionals.

Biochemical indicators are categorized based on the standardized reference ranges specified in the national community health examination form, and classified as negative, positive, normal, high, or low. See Table 1 for reference ranges and sources.

**Table 1:** Reference ranges and sources for biochemical indicators.

Biochemical indicators	Unit	Gender	Reference range	Reference sources
Hemoglobin (Hb)	g/L	Male	130–175	WS/T 406—2024 Clinical Blood Test Common Project Analysis Quality Standard
		Female	115–150	
White blood cells (WBC)	L	Male/female	3.50–9.50 × 10^9^	
Platelets (PLT)	L	Male/female	125–350 × 10^9^	
Red blood cells (RBC)	L	Male	4.0–5.5 × 10^12^	
		Female	3.5–5.0 × 10^12^	
Urine routine	/	/	“−” is negative, “+” is positive	/
Fasting blood glucose (FBG)	mmol/L	Male/female	3.89–6.11	China Diabetes Prevention and Control Guidelines (2020 Edition)
Alanine aminotransferase (ALT)	U/L	Male	9–50	WS/T 404 Clinical Common Biochemical Test Reference Interval
		Female	7–40	
Aspartate aminotransferase (AST)	U/L	Male	15–40	
		Female	13–35	
Total bilirubin (TBIL)	μmol/L	Male/female	3.4–20.5	
Creatinine (Cr)	μmol/L	Male	57–111	
		Female	41–81	
Blood urea nitrogen (BUN)	mmol/L	Male	3.6–9.5	
		Female	3.1–8.8	
Uric acid (UA)	μmol/L	Male/female	120–430	/
Total cholesterol (TC)	mmol/L	Male/female	<5.69	China Adult Dyslipidemia Prevention and Treatment Guidelines (2016 Revised Edition)
Triglycerides (TG)	mmol/L	Male/female	<1.70	
Low-density lipoprotein cholesterol (LDL-C)	mmol/L	Male/female	<3.64	
High-density lipoprotein cholesterol (HDL-C)	mmol/L	Male/female	0.9–2.0	

### 2.3 Inclusion of diseases

Based on a review of relevant literature and clinical guidelines, this study extracted data on 15 chronic conditions from residents’ health examination records: hypertension, fatty liver, dyslipidemia, diabetes, cataracts, heart disease, stroke, malignant tumors, chronic lung diseases, bone and joint diseases, chronic kidney diseases, spinal diseases, chronic digestive diseases, mental disorders, neurodegenerative diseases. Disease classification criteria: The first seven conditions were common chronic diseases; the remaining eight conditions, which were less prevalent, were categorized based on their clinical characteristics. Among them, fatty liver refers to liver lipid deposition diagnosed by doctors using ultrasound imaging (26). Dyslipidemia refers to TC ≥ 6.22 mmol/L, TG ≥ 2.26 mmol/L, LDL-C ≥ 4.14 mmol/L, HDL-C < 1.04 mmol/L, meeting any of the above (27).

### 2.4 Statistical analysis

Statistical analysis was performed using SPSS V.26.0. Binary logistic regression analysis was used to explore the association between multimorbidity of chronic diseases and biochemical indicators, and the forward stepwise Wald selection strategy was used in the regression model. In this study, *p* < 0.05 was considered statistically significant.

## 3 Results

### 3.1 Basic information of the population

Significant demographic differences were observed in multimorbidity patterns among the older adult population. The study population comprised 19,624 individuals, with 42.8% males and 57.2% females; 39.7% urban residents and 60.3% rural residents; 42.7% aged 60–70 years, 43.5% aged 70–80 years, and 13.9% aged 80 years and above.

The overall prevalence of multimorbidity was 70.3%. Statistically significant differences in multimorbidity rates were found across age groups (χ^2^ = 17.908, *p* < 0.001), with prevalence rates of 70.5% (5,904/8,369) in the 60–69 age group, 71.1% (6,072/8,538) in the 70–79 age group, and 66.9% (1,818/2,717) in those aged 80 and above. Significant gender differences in multimorbidity were observed, with females showing a higher prevalence (72.3%) compared to males (67.6%) (χ^2^ = 50.677, *p* < 0.001). Urban–rural differences were also notable, with urban residents showing significantly higher prevalence (78.9%) than rural residents (64.6%) (χ^2^ = 458.772, *p* < 0.001), as shown in Table 2. The relatively lower multimorbidity rate among those aged 80 and above might be attributed to survival bias or limited sample size in this age group, warranting further investigation.

**Table 2:** Basic information of older adult population.

Characteristics	Classification	Number	Percentage	Non-multimorbidity (*N* = 5,830)	Multimorbidity (*N* = 13,794)	Multimorbidity rate	*Χ*^2^ value
	Total	19,624	/	5,830	13,794	70.29%	/
Gender	Male	8,400	42.80%	2,721	5,679	67.60%	50.677***
	Female	11,224	57.20%	3,109	8,115	72.30%	
Age group	60	8,369	42.65%	2,465	5,904	70.50%	17.908***
	70	8,538	43.51%	2,466	6,072	71.10%	
	80	2,717	13.85%	899	1,818	66.90%	
Urban and rural	Urban	7,792	39.71%	1,644	6,148	78.90%	458.772***
	Rural	11,832	60.29%	4,186	7,646	64.60%	

*p < 0.05; **p < 0.01; ***p < 0.001.

### 3.2 Average multimorbidity numbers of chronic diseases

Among the 19,624 participants, the prevalence of chronic conditions was as high as 96.5%. Among the 15 chronic conditions studied, prevalence rates varied substantially, ranging from 0.1% for neurodegenerative diseases to 81.59% for hypertension. The top seven diseases in terms of prevalence are: hypertension (81.6%), fatty liver (46.9%), lipid abnormalities (42.0%), diabetes (19.8%), cataracts (7.2%), heart disease (7.1%), and stroke (6.9%).

The average number of multimorbidities in the total population was 2.15, with a standard deviation of 1.08. Among the top seven chronic conditions, hypertension, fatty liver, dyslipidemia, and diabetes had relatively lower average numbers of multimorbidities (<3), whereas cataracts, heart disease, and stroke had higher average numbers of multimorbidities (>3). This suggests that the multimorbidity patterns of these latter conditions are relatively complex, potentially requiring more comprehensive medical management strategies. Refer to Table 3 for details.

**Table 3:** Average multimorbidity numbers of chronic diseases.

Disease	Number of chronic diseases	Percentage (%)	Average multimorbidity numbers (x̅±s)
Total population	18,937	96.50	2.15 ± 1.08
Hypertension	16,011	81.59	2.31 ± 1.03
Fatty liver	9,197	46.87	2.82 ± 0.90
Dyslipidemia	8,233	41.95	2.87 ± 0.91
Diabetes	3,878	19.76	2.94 ± 1.10
Cataracts	1,411	7.19	3.33 ± 1.09
Heart disease	1,394	7.10	3.16 ± 1.16
Stroke	1,357	6.92	3.33 ± 1.17
Malignant tumor	231	1.18	3.20 ± 1.19
Chronic lung disease	198	1.01	2.88 ± 1.07
Bone and joint disease	109	0.56	3.24 ± 1.25
Chronic kidney disease	93	0.47	3.70 ± 1.32
Spinal disease	71	0.36	3.39 ± 1.24
Chronic digestive disease	43	0.22	2.60 ± 0.90
Mental disorders	32	0.16	3.53 ± 1.14
Neurodegenerative diseases	25	0.10	3.20 ± 1.12

### 3.3 Multimorbidity of chronic diseases

**Figure 1:**
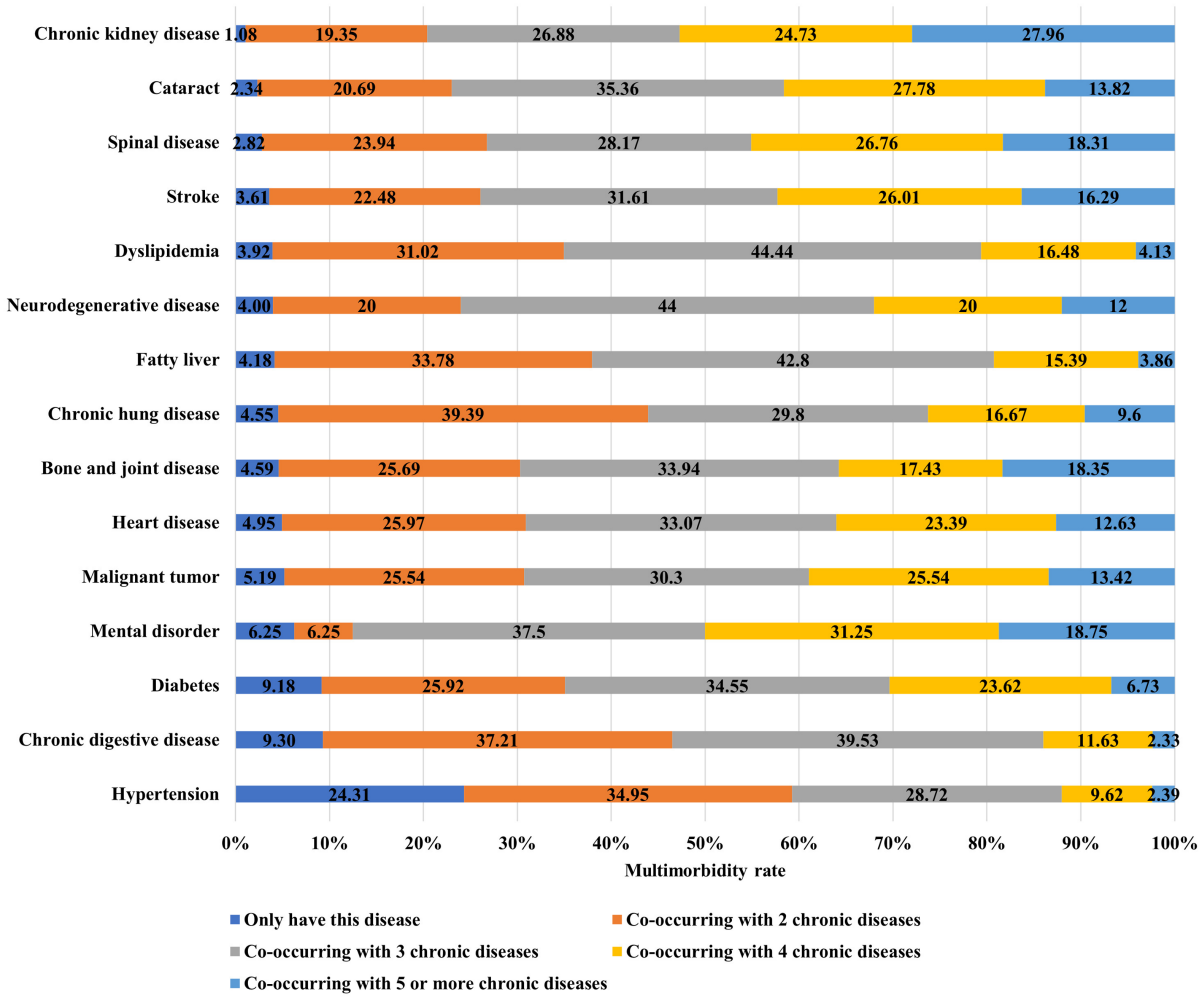
Disease and multimorbidity status.

In most cases of chronic diseases has one or more other chronic diseases, with a multimorbidity rate of over 90% for each chronic disease except hypertension. Specifically, among older adult people with hypertension, 24.3% only have hypertension, while 75.7% have one or more other chronic diseases. In older adult people with fatty liver, 95.8% have one or more other chronic diseases, while only 4.2% have fatty liver alone, as shown in Figure 1.

### 3.4 Correlation between multimorbidity of chronic diseases and biochemical indicators

Chi-square tests of biochemical indicators in older adult people shows statistically significant differences in the multimorbidity rates of chronic diseases for variables such as Hb, RBC, PLT, WBC, U-PRO, U-GLU, U-BLD, U-WBC, FBG, ALT, AST, Cr, UA, TC, TG, LDL-C, and HDL-C (*p* < 0.05), while there is no statistical difference in U-KET, TBIL, and BUN, as shown in Table 4.

**Table 4:** Correlation between multimorbidity of chronic diseases and biochemical indicators in older adult.

Indicator	Classification	Number	Percentage	Non-multimorbidity (*N* = 5,830)	Multimorbidity (*N* = 13,794)	Multimorbidity rate	*p* value
Hb	Normal	16,761	85.41%	4,928	11,833	70.60%	<0.001
	Low	2,309	11.77%	813	1,496	64.79%	
	High	554	2.82%	89	465	83.94%	
RBC	Normal	18,576	94.66%	5,516	13,060	70.31%	<0.001
	Low	360	1.83%	173	187	51.94%	
	High	688	3.51%	141	547	79.51%	
PLT	Normal	18,391	93.72%	5,451	12,940	70.36%	0.018
	Low	862	4.39%	285	577	66.94%	
	High	371	1.89%	94	277	74.66%	
WBC	Normal	17,646	89.92%	5,237	12,409	70.32%	<0.001
	Low	1,208	6.16%	455	753	62.33%	
	High	770	3.92%	138	632	82.08%	
U-PRO	−	13,556	69.08%	4,248	9,308	68.66%	<0.001
	+	6,068	30.92%	1,582	4,486	73.93%	
U-GLU	−	17,993	91.69%	5,561	12,432	69.09%	<0.001
	+	1,631	8.31%	269	1,362	83.51%	
U-KET	−	18,776	95.68%	5,603	13,173	70.16%	0.055
	+	848	4.32%	227	621	73.23%	
U-BLD	−	14,537	74.08%	4,174	10,363	71.29%	<0.001
	+	5,087	25.92%	1,656	3,431	67.45%	
U-WBC	−	12,778	65.11%	3,922	8,856	69.31%	<0.001
	+	6,846	34.89%	1,908	4,938	72.13%	
FBG	Normal	13,711	69.87%	4,683	9,028	65.84%	<0.001
	Low	172	0.88%	47	125	72.67%	
	High	5,741	29.25%	1,100	4,641	80.84%	
ALT	Normal	18,397	93.75%	5,635	12,762	69.37%	<0.001
	Low	84	0.43%	25	59	70.24%	
	High	1,143	5.82%	170	973	85.13%	
AST	Normal	17,678	90.08%	5,408	12,270	69.41%	<0.001
	Low	85	0.43%	18	67	78.82%	
	High	1,861	9.48%	404	1,457	78.29%	
TBIL	Normal	16,717	85.19%	4,929	11,788	70.52%	0.100
	High	2,907	14.81%	901	2,006	69.01%	
Cr	Normal	16,287	83.00%	5,033	11,254	69.10%	<0.001
	Low	185	0.94%	61	124	67.03%	
	High	3,152	16.06%	736	2,416	76.65%	
BUN	Normal	17,949	91.46%	5,344	12,605	70.23%	0.771
	Low	133	0.68%	37	96	72.18%	
	High	1,542	7.86%	449	1,093	70.88%	
UA	Normal	16,356	83.35%	5,080	11,276	68.94%	<0.001
	Low	51	0.26%	19	32	62.75%	
	High	3,217	16.39%	731	2,486	77.28%	
TC	Normal	15,025	76.56%	5,031	9,994	66.52%	<0.001
	High	4,599	23.44%	799	3,800	82.63%	
TG	Normal	7,799	39.74%	3,513	4,286	54.96%	<0.001
	High	11,825	60.26%	2,317	9,508	80.41%	
LDL-C	Normal	16,990	86.58%	5,493	11,497	67.67%	<0.001
	High	2,634	13.42%	337	2,297	87.21%	
HDL-C	Normal	15,850	80.77%	4,841	11,009	69.46%	<0.001
	Low	1,084	5.52%	46	1,038	95.76%	
	High	2,690	13.71%	943	1,747	64.94%	

### 3.5 Logistic regression analysis of factors influencing biochemical indicators of multimorbidity

Variables with statistically significant differences, including Hb, RBC, PLT, WBC, U-PRO, U-GLU, U-BLD, U-WBC, FBG, ALT, AST, Cr, UA, TC, TG, LDL-C, and HDL-C, were incorporated into binary logistic regression analysis. After adjusting for gender, age, and place of residence, the results showed that elevated levels of Hb, FBG, ALT, AST, Cr, and UA were associated with a higher risk of multimorbidity (*p* < 0.05), while reduced levels did not show statistical differences. High WBC, high RBC, positive U-PRO, positive U-GLU, high TC, high TG, high LDL-C, low HDL-C are also correlated with a higher risk of multimorbidity of chronic diseases (*p* < 0.05). Additionally, low WBC, low RBC, and positive U-BLD are associated with a lower risk of multimorbidity. Refer to Table 5 for details. A total of 14 statistical tests were conducted in this study. After applying Bonferroni adjustment, the significance level was set at *p* < 0.0036. Following this adjustment, 12 of the test results remained significant.

**Table 5:** Logistic regression analysis of factors influencing biochemical indicators of multimorbidity in older adult.

Indicator	*B* value	Sx	Wald *Χ*^2^ value	*p* value	Exp(B)	95%CI	
**Hb**			10.423	0.005			
Low	−0.086	0.064	1.801	0.180	0.917	0.809	1.041
High	0.380	0.132	8.278	0.004	1.462	1.129	1.895
**WBC**			16.247	<0.001			
Low	−0.396	0.117	11.422	0.001	0.673	0.535	0.847
High	0.223	0.105	4.512	0.034	1.250	1.017	1.536
**RBC**			12.694	0.002			
Low	−0.171	0.085	4.060	0.044	0.843	0.714	0.995
High	0.309	0.109	8.050	0.005	1.362	1.100	1.687
**U-PRO**	0.096	0.039	6.158	0.013	1.101	1.020	1.187
**U-GLU**	0.361	0.077	21.758	<0.001	1.435	1.233	1.670
**U-BLD**	−0.183	0.039	21.420	<0.001	0.833	0.771	0.900
**FBG**			220.704	<0.001			
Low	0.188	0.186	1.022	0.312	1.207	0.838	1.737
High	0.636	0.043	220.594	<0.001	1.889	1.737	2.054
**ALT**			26.296	<0.001			
Low	0.196	0.270	0.525	0.469	1.216	0.716	2.064
High	0.536	0.106	25.758	<0.001	1.708	1.389	2.101
**AST**			6.958	0.031			
Low	−0.122	0.311	0.155	0.694	0.885	0.481	1.626
High	0.195	0.075	6.786	0.009	1.216	1.050	1.408
**Cr**			23.644	<0.001			
Low	−0.177	0.176	1.012	0.314	0.838	0.593	1.183
High	0.250	0.053	22.426	<0.001	1.284	1.158	1.424
**UA**			33.476	<0.001			
Low	0.008	0.321	0.001	0.980	1.008	0.538	1.890
High	0.305	0.053	33.473	<0.001	1.357	1.224	1.505
**TC**	0.564	0.052	116.636	<0.001	1.758	1.587	1.947
**TG**	0.967	0.035	754.428	<0.001	2.631	2.456	2.819
**LDL-C**	0.610	0.072	72.618	<0.001	1.840	1.599	2.117
**HDL-C**			241.972	<0.001			
Low	2.398	0.155	239.746	<0.001	10.999	8.120	14.900
High	−0.041	0.049	0.699	0.403	0.960	0.871	1.057

## 4 Discussion

In this study, hypertension was the most prevalent chronic condition at 81.6%, consistent with previous research (28). Notably, fatty liver (46.9%) and dyslipidemia (42.0%) ranked second and third, respectively; their high prevalence may reflect the metabolic characteristics and lifestyle changes among the older adult population. Regarding multimorbidity, among the 15 chronic conditions studied, the proportion of patients with other concurrent chronic conditions ranged from 75.7 to 98.9%, indicating that chronic conditions rarely exist in isolation.

The prevalence of multimorbidity among the older adult in this study was 70.3%, which differs from existing literature. Several domestic studies have reported multimorbidity rates between 40 and 50% among Chinese older adult (17–19), while some rural areas have reported rates exceeding 90% (20). In developed countries such as Spain and Germany, the prevalence of multimorbidity among the older adult is approximately 60% (29, 30). These significant variations in multimorbidity rates across studies may be attributed to multiple factors, including geographical environment, recruitment methods, sample size, data collection methods, and differences in multimorbidity definitions (31). Therefore, in-depth multimorbidity research targeting older adult populations in specific regions has important epidemiological significance, contributing to a more precise understanding of regional multimorbidity patterns and health management strategies.

Biochemical indicators are important clinical tools widely used in routine health examinations, disease screening, treatment decisions, and therapeutic monitoring. Our results demonstrate that Hb, FBG, ALT, AST, Cr, and UA were associated with multimorbidity risk only when their levels were elevated. Notably, HDL-C, known as ‘good cholesterol’, showed a pattern different from other indicators, with lower levels associated with higher multimorbidity risk. Our findings partially align with existing literature, such as the association between ALT and multimorbidity (24) and the relationship between WBC and multimorbidity (25). Compared to other indicators in this study, elevated Hb, high FBG, positive U-GLU, elevated ALT, and abnormal blood lipids showed the most significant associations, with multimorbidity risk increasing by nearly 1.5-fold.

Hb, as a key protein responsible for oxygen transport in red blood cells (32), may reflect various physiological and pathological conditions, including hypoxic compensation, when its levels are abnormal. In this study, we observed that elevated Hb levels in older adult individuals were associated with a significant 1.462-fold increase in multimorbidity risk. Existing research suggests that abnormal Hb levels may be related to multiple physiological processes: elevated Hb can increase blood viscosity and peripheral vascular resistance, slow blood circulation, reduce effective transport of oxygen and metabolites, and delay the clearance of metabolic waste (33). Previous studies have indicated that higher Hb levels may be associated with adverse metabolic states and mortality (34, 35). Some studies have also found potential associations between Hb levels and cardiovascular mortality (36), rheumatoid arthritis (37), and non-alcoholic fatty liver disease (38). From a pathophysiological perspective, abnormal Hb levels may contribute to the development of multimorbidity through mechanisms including endothelial dysfunction, activation of inflammatory responses, and increased oxidative stress.

FBG is the most commonly used indicator for diabetes, reflecting the body’s insulin regulation function for blood glucose. The “glycotoxicity” of high blood glucose damages insulin secretion (39), and it is also an significant cause of dysfunction in vascular endothelial cells. Hyperglycemia induces oxidative stress, which further promotes inflammation in cells with an inflammatory background, ultimately leading to vascular damage (40). Previous studies have found associations between FBG and various vascular diseases, including hypertension, myocardial infarction, and stroke (41–44), as well as cognitive decline (45) and elevated uric acid levels (46). In this study, we observed that 29.3% of the older adult population had elevated FBG levels, and this elevation was statistically associated with a 1.889-fold increase in multimorbidity risk. This finding provides important insights for future research on chronic disease multimorbidity management in older adult populations.

The presence of U-GLU is primarily determined by three factors: blood glucose levels, the glomerular filtration rate of the kidneys concerning blood glucose, and the reabsorption capacity of glucose by the renal tubules. The presence of U-GLU can be categorized into two major categories: high blood glucose with U-GLU and normal blood glucose with U-GLU. The harm caused by the presence of U-GLU is primarily attributed to the biological effects of cellular damage and dysfunction under high blood glucose conditions (47). Prolonged high blood glucose levels may lead to other complications such as cardiovascular disease, kidney disease, neurological disorders, and retinopathy (48). In this study, we observed that individuals with positive U-GLU had a 1.44-fold increase in multimorbidity risk, providing preliminary epidemiological evidence for understanding the relationship between positive U-GLU and multimorbidity in older adult populations.

ALT, one of the sensitive markers of liver injury, is primarily distributed in hepatic cells (49), and its abnormal levels may reflect various complex physiological and pathological processes. Previous studies have widely reported associations between ALT and all-cause mortality risk (50, 51), as well as various chronic conditions, including fatty liver (52), hypertension (53), type 2 diabetes (54), cardiovascular diseases (49, 55), and metabolic syndrome (56). Prior research suggests that elevated ALT levels are independently associated with multimorbidity in a dose–response manner, with insulin resistance, chronic inflammatory response, and liver enzyme synthesis disorders being potential underlying mechanisms (24). In this study, we observed that 5.82% of older adult individuals had elevated ALT levels, and this elevation was statistically associated with a 1.708-fold increase in multimorbidity risk, providing preliminary evidence for further exploration of its value as a potential biomarker.

Among the lipid indicators, TC, TG, and LDL-C have a role in atherosclerosis, with their excessive accumulation associated with various chronic diseases including cardiovascular diseases, type 2 diabetes, obesity, and non-alcoholic fatty liver disease (57). However, the principal function of HDL-C is the metabolism and excretion of cholesterol. In addition to its anti-atherosclerotic properties, HDL-C also demonstrates anti-inflammatory, antioxidant, anti-diabetic, and fibrinolytic characteristics (58, 59). Previous studies have demonstrated associations of HDL-C with insulin resistance, dyslipidemia, atherosclerosis index, and obesity (60). Low HDL-C is significantly associated with increased risks of diabetes, obesity, and hypertension (61), and is an important consideration in assessing coronary heart disease risk (59). In this study, among the older adult population, the proportions of elevated TC, TG, and LDL-C were 23.4, 60.3, and 13.4%, respectively, and these elevations were statistically associated with increased multimorbidity risk by 1.758-fold, 2.631-fold, and 1.840-fold, respectively. Low HDL-C levels were observed in 5.5% of the population and were significantly associated with multimorbidity risk, showing a nearly 11-fold increase. These findings suggest that blood lipid indicators may be important entry points for understanding the potential mechanisms of multimorbidity, though further research is needed to elucidate their intrinsic connections.

This study systematically analyzed the associations between various biochemical indicators and multimorbidity in older adult populations. These indicators may interact through two levels of biological mechanisms: first, through common pathophysiological processes (such as systemic inflammation, oxidative stress, and endothelial dysfunction); and second, through individual heterogeneity factors (such as age, gender, and urban–rural differences) that influence disease susceptibility. These findings reveal the significant value of monitoring specific biochemical indicators in managing multimorbidity among the older adult, providing crucial insights for future research. Future research directions include: (1) elucidating the biological mechanisms linking abnormal indicators and multimorbidity; (2) evaluating the clinical value of these indicators in multimorbidity risk screening; (3) exploring dose–response relationships between indicators and disease risk; and (4) conducting prospective studies to validate their predictive significance. These studies will contribute to developing a biochemical indicator-based system for multimorbidity assessment and management, thereby promoting healthy aging.

This study has several limitations. First, some disease data rely on self-reports from residents, which may lead to recall bias and misclassification of diseases, thereby affecting the accurate identification of multimorbidity and the assessment of prevalence rates. Second, the selection of only 15 diseases to define multimorbidity may result in an underestimation of prevalence, impacting the association between multimorbidity and biochemical indicators. Additionally, the low reporting rates of non-conventional screening diseases, particularly mental health conditions, limit the generalizability of the results, and the lack of information on socioeconomic background and lifestyle factors may also affect external validity. To address these limitations, we recommend: (1) use standardized disease diagnostic tools and establish standardized health record systems (2) designing comprehensive survey questionnaires to systematically collect socioeconomic indicators; (3) adopting standardized lifestyle assessment tools; (4) expanding disease spectrum screening, especially including mental health conditions, to improve data comprehensiveness and result generalizability; and (5) conducting prospective cohort studies to establish dynamic association models. Despite these limitations, this study provides important preliminary evidence for multimorbidity in older adult populations.

## 5 Conclusion

Through a systematic analysis of the older adult population in Jindong District, Jinhua City, this study provides a detailed profile of multimorbidity in community-dwelling older adult at the municipal level. The study found that the multimorbidity rate among the older adult in this region reached 70.3%, with the main disease burden being hypertension and fatty liver, each affecting over 40% of the population. The research revealed significant associations between specific biochemical indicators (HDL-C, TG, FBG, ALT, U-GLU, and Hb) and multimorbidity. These findings provide important evidence for establishing risk assessment and health management systems for multimorbidity among older adult populations in primary care communities.

## Data Availability

The raw data supporting the conclusions of this article will be made available by the authors, without undue reservation.
